# Long noncoding RNA LINC00152 promotes cell proliferation through competitively binding endogenous miR‐125b with MCL‐1 by regulating mitochondrial apoptosis pathways in ovarian cancer

**DOI:** 10.1002/cam4.1547

**Published:** 2018-07-20

**Authors:** Puxiang Chen, Xiaolin Fang, Bing Xia, Yan Zhao, Qiaoyan Li, Xiaoying Wu

**Affiliations:** ^1^ Department of Gynecology and Obstetrics The Second Xiangya Hospital of Central South University Changsha China; ^2^ Hunan Cancer Hospital Changsha China; ^3^ Department of Gynecology and Obstetrics The Maternal and Child Health Hospital of Hunan Province Changsha China; ^4^ Department of Gynecology and Obstetrics The Third Xiangya Hospital of Central South University Changsha China; ^5^ Department of Pathology Xiangya Hospital Central South University Changsha China; ^6^ Department of Pathology School of Basic Medical Science Central South University Changsha China

**Keywords:** cell proliferation, competitive endogenous RNA, LINC00152, long noncoding RNA, MCL‐1, MiR‐125b, mitochondrial apoptosis pathways, ovarian cancer

## Abstract

Recently, an increasing number of studies have focused on the key function of long noncoding RNAs (lncRNAs) in biological activity. Abnormal lncRNA expression was found to relate to the development and pathogenesis of multiple cancers. LncRNA LINC00152 served as an oncogene in multiple cancers; however, its role in ovarian cancer remains unknown. In our research study, LINC00152 was upregulated in ovarian cancer tissues and cell lines. An increasing LINC00152 level was positively correlated with the histological grade, clinical stage, and poor prognosis of ovarian cancer patients. In addition, knockdown of LINC00152 reduced cell growth, induced cell apoptosis, and suppressed tumor growth. Moreover, we revealed that LINC00152 and Myeloid cell leukemia‐1 (MCL‐1) were targeted by miR‐125b and had the same miR‐125b combining site. The miR‐125b level was negatively correlated with the expression of LINC00152, while MCL‐1 was positively related to the LINC00152 level. MiR‐125b could affect LINC00152 levels as evaluated by qRT‐PCR. Finally, we affirmed that LINC00152 mediated cell proliferation by affecting MCL‐1 expression and MCL‐1‐mediated mitochondrial apoptosis pathways and by working as a competitive endogenous RNA (ceRNA) of miR‐125b. In summary, based on ceRNA theory, the combined research on miR‐125b and MCL‐1, and taking LINC00152 as a new study point, we provide new insight into the molecular mechanism of reversing cell proliferation in ovarian cancer.

## 1 INTRODUCTION

Ovarian cancer is a common gynecological malignant tumor, as well as one of the most lethal gynecological malignancies in the world.^1^ Despite improvements in surgery, radiotherapy and neoadjuvant chemotherapy as the main methods to treat ovarian cancer, the 5‐year survival rate of patients is still only 30%.^2^ The lack of biomarkers for early detection and diagnosis is the major cause of the low survival rate. Poor understanding of the mechanism of development of ovarian cancer also contributes to high mortality.

Recent studies have shown that long noncoding RNAs (lncRNAs), a class of noncoding RNA transcripts of 200 nucleotides in length, can distinguish markedly between ovarian carcinoma tissue and corresponding normal tissue.^3^ LncRNAs frequently function as tumor suppressors or oncogenes. For example, a low level of lncRNA GAS5 produced a poorer overall survival rate against ovarian cancer. Furthermore, upregulation of GAS5 inhibits ovarian cancer growth through regulating apoptosis protease activating factor 1 (APAF1), cyclin D1, and p21 levels.^4^ The antisense lncRNA HOXA11 had higher expression levels in ovarian cancer tissues than compared to normal tissues, as well as a positive correlation with histological grade, suggesting that it is a prognostic factor of progressive disease and mortality. HOXA11 antisense lncRNA overexpression in ovarian cancer promoted cell proliferation, invasion, and migration by regulating the epithelial‐mesenchymal transition (EMT).^5^ Therefore, lncRNAs are expected to be new markers and targets for the diagnosis and treatment of malignant ovarian tumors.

LINC00152, an 828‐bp lncRNA, is located at chromosome 2p11.2. It was initially detected as differentially hypomethylated during hepatocarcinogenesis.^6^ LINC00152 also served as an oncogene in cancer, including hepatocellular carcinoma, clear cell renal cell carcinoma, gastric cancer, and gallbladder cancer.^7^, ^8^, ^9^ In hepatocellular carcinoma, overexpression of LINC00152 was dramatically promoted during tumor growth by activation of the mTOR signaling pathway.^10^ In clear cell renal cell carcinoma, a high LINC00152 expression level was closely associated with advanced clinical stage and served as an independent predictor of overall prognosis and survival.^11^ MicroRNAs (miRNAs) are 20‐200 nucleotides in length and function as an oncogene or tumor suppressor through regulating target genes via interfering with transcription or inhibiting translation.^12^ MiR‐125b has been reported to decrease in ovarian cancer tissues and cell lines, and therefore, upregulation of miR‐125b could inhibit ovarian cancer growth through targeting BLC‐3.^13^ MicroRNA‐125b was targeted by Mcl‐1, Bcl‐w, and IL‐6R to promote cancer cell apoptosis.^14^ In addition, miR‐125b could modulate oxygen consumption and mitochondrial gene expression.^15^ Overexpression of miR‐125b enhanced the cytotoxicity of doxorubicin in doxorubicin‐resistant MCF‐7 cells. This sensitization of miR‐125b is caspase dependent and leads to a loss of mitochondrial membrane potential and mitochondrial outer membrane permeability.^16^ Furthermore, miR‐125b‐mediated mitochondrial apoptosis pathways also play important roles in chemotherapy drug‐induced apoptosis.^17^, ^18^, ^19^ The competitive endogenous RNA (ceRNA) theory for miRNA and lncRNA is currently widely accepted.^20^ To date, the role of LINC00152 in ovarian cancer is unclear, and whether LINC00152 achieves its role in cancer through the miR‐125b‐mediated mitochondrial pathway remains unclear.

Considering its crucial role in the pathogenesis of the cancers mentioned above, we aimed to research the function of LINC00152 in ovarian cancer development. In this study, we showed that LINC00152 was upregulated in ovarian cancer tissues and cell lines, and knockdown of LINC00152 inhibits cell proliferation, induces apoptosis in vitro, and suppresses tumor growth in vivo, by acting as a ceRNA of miR‐125b to regulate its targets within the MCL‐1‐mediated mitochondrial pathway. Thus, LINC00152 may be a novel molecule involved in ovarian cancer progression, as well as a potential prognostic biomarker and therapeutic target.

## 2 MATERIALS AND METHODS

### 2.1 Tissue samples, cell lines, and cell transfection

Ovarian cancer tissue chips, including 82 cases of ovarian cancer tissues and 14 cases of normal ovarian tissues, were purchased from Auragene (Changsha, China). Twenty ovarian cancer tissues and their corresponding normal tissues were collected from patients who underwent surgical resections at the Second Xiangya Hospital of Central South University (Changsha, China), snap‐frozen in liquid nitrogen, and then stored at −80°C until further use. This project was approved by the Ethics Committee of The Second Xiangya Hospital of Central South University.

The normal human ovarian epithelial cells (HOSE) and human ovarian cancer cell lines, including HO‐8910, SKOV3 and A2780, were obtained from ATCC. Cells were grown routinely in RPMI‐1640 medium (Invitrogen, CA, USA) supplemented with 10% fetal bovine serum (Gibco, CA, USA) and cultured in a 37°C humidified atmosphere of 5% CO_2_. Overexpression of miR‐125b and silencing of miR‐125b in SKOV3 and A2780 cells were achieved by transfection with either miR‐125b mimics or miR‐125b inhibitor (Genepharma, Shanghai, China) using Lipofectamine 6000 (Invitrogen, CA, USA). Knockdown of LINC00152 and overexpression of LINC00152 cell models were established by transfecting LINC00152 shRNA (GeneCopoecia, Guangzhou, China) or pcDNA3.1‐LINC00152 (GeneCopoecia, Guangzhou, China) into SKOV3 and A2780 cells for 48 hours; the cells that were transfected with pcDNA3.1 were used as negative control.

### 2.2 In situ hybridization

The in situ hybridization (ISH) probe that was used for detecting LINC00152 levels by labeling with digoxin was designed and synthesized by Sangon Biotech Co., Ltd. (Shanghai, China) (5′‐CTTCATTGAACA GTTTGTATATTGGAAACTTGCC‐3′). The method of the ISH experiment was performed as described by Luo et al^21^ Stained images were taken with a light microscope (magnification 200× and 400×, AE31, MOTIC CHINA GROUP CO., LTD, China).

### 2.3 RNA extraction and SYBR green quantitative PCR analysis

TRIzol reagent (Invitrogen, CA, USA) was used for extracting total RNA from cells and tissues. A Hairpin‐it TM miRNAs qPCR kit (Genepharma, Shanghai, China) was used to measure miR‐125b levels according to the manufacturer's instructions. The expression of LINC00152 was assessed by SYBR green qRT‐PCR assay (Takara, Dalian, China), and β‐actin was used as an endogenous control. The primers were used as follows: LINC00152, sense, TGAGAATGAAGGCTGAGGTGT, antisense, GCAGCGACCATCCAGTCATT; β‐actin, sense, AGGGGCCGGACTCGTCATACT, antisense, GGCGGCACCACCATGTACCCT. The primers of miR‐125b and U6 were obtained from FUNENG(Guangzhou, China). qRT‐PCR was performed at the following conditions: 95.0°C for 3 minutes and 39 circles of 95.0°C for 10 seconds followed by 60°C for 30 seconds. Data were processed using 2^−ΔΔCT^ method.

### 2.4 CCK‐8 cell proliferation assay

Cell proliferation rates were measured using the Cell Counting Kit‐8 (CCK‐8) (Beyotime, Hangzhou, China). A total of 5 × 10^3^ cells were seeded in each 96‐well plate for 24 hours, transfected with the indicated plasmids, and further incubated for 24, 48 or 72 hours. Ten microliters of CCK‐8 reagents were added to each well 1 hours before the endpoint of the incubation period. The OD 450 nm absorbance value in each well was determined by a microplate reader.

### 2.5 Flow cytometric analysis

An annexin V apoptosis detection kit (Life technologies, Grand Island, NY) was used for analysis of apoptosis. Approximately 2 × 10^5^ cells were harvested, washed twice with cold phosphate‐buffered saline (PBS), and then resuspended in 500 μL of binding buffer. Ten microliters of Annexin V‐FITC and 10 μL of propidium iodide were added to the solution and mixed well. After 15 minutes of incubation, the cells were analyzed using flow cytometry (BD Biosciences, San Jose, CA).

### 2.6 Hoechst 33258 staining assay

For the Hoechst 33258 staining assay, the Hoechst 33258 staining kit (Beyotime, Shanghai, China) was used to observe the apoptotic cells and measure the apoptosis ratio of cancer cells at 48 hours.^22^

### 2.7 Western blot analysis

Cultured or transfected cells were lysed in RIPA buffer with 1% PMSF. Protein was loaded onto a SDS‐PAGE minigel and transferred onto a PVDF membrane. After being probed with primary antibody (anti‐Mcl‐1, anti‐Bcl‐2, anti‐Bax, anti‐cytochrome *c*, anti‐cleaved caspase 9, anti‐cleaved caspase 3, and anti‐cleaved PARP from ImmunoWay Biotechnology Company, Plano, TX, USA; anti‐Bcl‐2 from ImmunoWay Biotechnology Company, Plano, TX, USA; and anti‐β‐actin from Abcam, Cambridge, UK) at 4°C overnight, the blots were subsequently incubated with secondary antibody (1:5000, Auragene, Changsha, China). Signals were visualized using ECL Substrates (Millipore, MA, USA). β‐actin was used as an endogenous protein for normalization. The results were assessed by IPP6.0 software.

### 2.8 Luciferase reporter assay

The fragment of LINC00152 (NCBI Reference Sequence: NR_027451.1) containing the putative miR‐125b binding site and mutant seed sequence of miR‐125b was synthesized by Genepharma Co., Ltd. (Shanghai, China) into a psiCHECK‐2 vector. Native and mutated MCL‐1 3′UTR was synthesized by Genepharma Co., Ltd. (Shanghai, China). All constructs were verified by DNA sequencing. The cells were plated in 96‐well clusters and then cotransfected with 100 ng of constructs with or without pre‐miR‐125b. At 48 hours after transfection, luciferase activity was detected using a dual‐luciferase reporter assay system (Promega, Madison, WI) and normalized to Renilla activity.

### 2.9 RNA immunoprecipitation (RIP)

RNA Immunoprecipitation experiments were performed using the Magna RIP RNA‐Binding Protein IP Kit (Millipore, Bedford, MA, USA) and the Ago2 antibody (Cell Signaling, Danvers, MA, USA) according to the manufacturer's instructions. Finally, purified RNAs in the precipitate fractions were used to determine LINC00152 and miR‐125b expression.

### 2.10 In vivo tumorigenicity assay

To determine tumor growth in vivo, SKOV3 cell lines transfected with sh‐LINC00152 and/or anti‐miR‐125b were suspended in culture medium at a density of 1 × 10^7^ cells/mL. BALB/c mice were acquired at 4 weeks old (Hunan SJA Laboratory Animal Co., certificate # No.43004700016031) and kept under 10 (light)/14 (dark) hours cycle environment with free access to food and water. Cells were inoculated subcutaneously (4 × 10^6^ cells/injection) into the axilla area of the mice (3 mice per group). The growth of tumors was monitored every 3 days, and the body weight and tumor size of the mice were measured and recorded (a nodule with a diameter longer than 0.4 cm was considered a tumor). The tumor volume was calculated with the following equation: *V* = A*B ^2^/2 (mm^3^). At the end of the experiment, the mice were decapitated, and tumors were isolated for further measurement.

### 2.11 Statistical analysis

All data from 3 independent experiments were expressed as the mean ± SD and processed using SPSS17.0 statistical software. The overall survival rate estimates over time were calculated using the Kaplan‐Meier method with a log‐rank test. The clinical association between LINC00152 expression and clinic‐pathological variables in ovarian cancer patients was evaluated by chi‐square test. The difference among the groups was estimated by Student's *t*‐test (2 groups) or one‐way ANOVA (more than 2 groups), depending on the conditions. *P value of <.05 was considered statistically significant.

## 3 RESULTS

### 3.1 LINC00152 is upregulated in ovarian cancer tissues and cell lines

**Figure 1:**
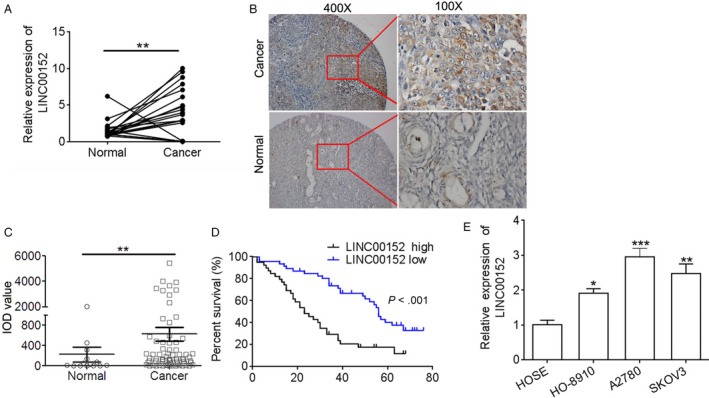
LINC00152 is upregulated in ovarian cancer tissues and cell lines. A, qRT‐PCR was performed to detect the expression of LINC00152 in paired normal and ovarian cancer tissues (n = 20). B, ISH was performed to detect LINC00152 expression in ovarian cancer tissues and normal ovarian tissues; magnification, 100× (left), 400× (right). C, Quantification of ISH staining. IOD, integral optical density. D, Survival cure showed that patients with low LINC00152 had a better survival rate than those of with high LINC00152 expression. E, qPCR was performed to detect the expression of LINC00152 in ovarian cancer cell lines (HO‐8910, A2780, and SKOV3) and normal human ovarian epithelial cells (HOSE). Error bars represent the mean ± SD of triplicate experiments, *P <* *.05, **P <* *.01, ***P <* *.001

To assess the LINC00152 level in ovarian cancer tissues, qRT‐PCR and ISH assays were performed. The results showed that LINC00152 expression was significantly increased in ovarian cancer tissues in comparison with normal ovarian tissues (Figure 1A‐C) and was localized to the cytoplasm (Figure 1B). Moreover, LINC00152 expression was positively associated with ovarian cancer histological grade, clinical stage (Table 1) and low survival rate (Figure 1D,E, *P* < .01). In addition, we measured the LINC00152 level in ovarian cancer cells and normal cells by qRT‐PCR assay. The results revealed that the LINC00152 level was frequently increased in ovarian cancer cell lines (HO‐8910, A2780, SKOV3) in comparison with the control human ovarian epithelial cells (HOSE) (Figure 1F). Collectively, these data suggest that the lncRNA LINC00152 may function as an oncogene during carcinogenesis of ovarian cancer.

**Table 1:** The association between LINC00152 expression and clinicopathological features of patients with ovarian cancer

Clinicopathological features	No cases	LINC00152 expression		*P* value
		Low	High	
Age (y)				
>45	43	22	21	0.833
≤45	39	18	21	
Grade				
I	14	9	5	
II	32	20	12	0.028^a^
III + IV	36	12	24	
T				
1	23	17	6	0.024^a^
2	14	5	9	
3	45	19	26	

a *P *< 0.05.

### 3.2 Downregulation of LINC00152 inhibits cell proliferation of ovarian cancer cells

**Figure 2:**
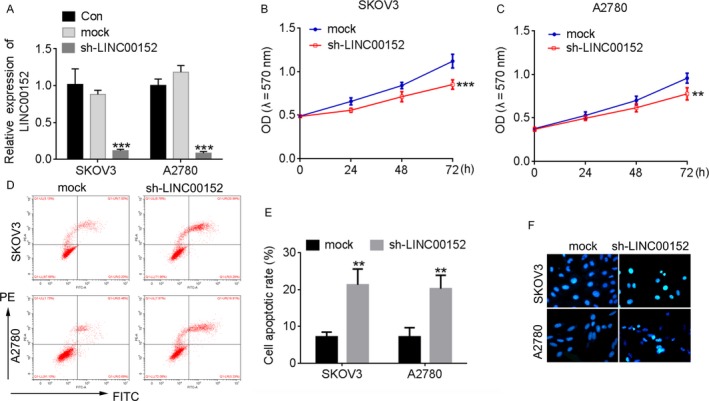
Downregulation of LINC00152 inhibits the cell proliferation of ovarian cancer cells. A, qRT‐PCR was performed to detect the expression of LINC00152 in SKOV3 and A2780 cells after transfection with sh‐LINC00152. The cells treated with sh‐NC were used as control. B,C, Knockdown of LINC00152 inhibited the proliferation of SKOV3 (B) and A2780 (C) cells detected by CCK8 assay. D, Downregulation of LINC00152 induced an increased apoptosis rate inSKOV3 and A2780 cells. E, Quantification of the apoptosis rate in (D). F, Hoechst 33258 stain assay detected the apoptosis cells in LINC00152Silencingof SKOV3 and A2780 cells. Error bars represent the mean ± SD of triplicate experiments, **P <* *.01

The biological role of LINC00152 in ovarian cancer remains unclear. Therefore, we used loss‐of‐function studies to investigate the function of LINC00152 in SKOV3 and A2780 cells. First, we detected the LINC00152 expression level in a loss‐of‐function model (Figure 2A). Then, the effect of LINC00152 on the proliferation of ovarian cancer was assessed using the CCK8 assay, and the apoptosis rate was detected by flow cytometry and Hoechst 33258 stain assay. The CCK8 results revealed that downregulation of LINC00152 dramatically impeded the cell proliferation in SKOV3 and A2780 cells (Figure 2B,C). A corresponding effect on apoptosis was also observed in a flow cytometry assay, which showed that knockdown of LINC00152 dramatically induced apoptosis in SKOV3 and A2780 cells compared with the control group (Figure 2D,E). In addition, we also found that LINC00152 downregulation dramatically increased apoptotic cells (Figure 2F). These results indicated that knockdown of LINC00152 inhibited cell growth and promoted cell apoptosis in ovarian cancer.

### 3.3 LINC00152 is a target of miR‐125b and modulates its target MCL‐1

**Figure 3:**
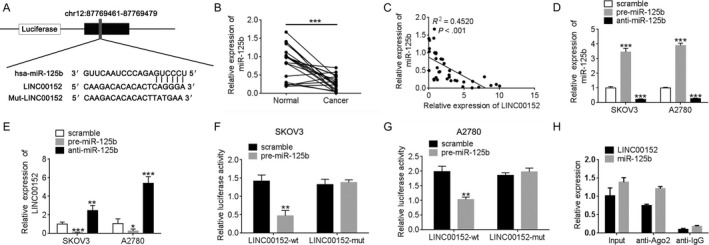
Identification of miR‐125b as a target of LINC00152. A, Alignment of potential LINC00152 sequences with miR‐125b as identified by miRcode (http://www.mircode.org). B, qRT‐PCR was performed to detect the expression of miR‐125b in paired normal and ovarian cancer tissues (n = 20). C, The correlation between theLINC00152 expression level and miR‐125b level was measured in 20 ovarian cancer tissues (*P* < .001). D, miR‐125b is downregulated and overexpression in SKOV3 and A2780cells compared to control groups as determined by qRT‐PCR. The expression of miR‐125b was normalized to U6. E, The expression of LINC00152 was upregulated after silencing miR‐125b in SKOV3 and A2780 cells. F,G, The relative luciferase activities were inhibited in the SKOV3 (F) and A2780 (G) cells cotransfected with wild‐type LINC00152 vector and pre‐miR‐125b, and not with the mutant‐type vector. Firefly luciferase activity was normalized to Renilla luciferase. H, Association of LINC00152 and miR‐125b with Ago2 in SKOV3 cells. Cellular lysates from SKOV3 cells were used for RIP with antibody against Ago2. LINC00152 and miR‐125b expression levels were detected using qRT‐PCR. Error bars represent the mean ± SD of triplicate experiments, *P <* *.05, **P <* *.01, ***P <* *.001

Starbase V2.0 and miRcode were used to predict potential miRNAs bound to LINC00152 and showed that miR‐125b has a binding site for LINC00152 (Figure 3A). Then, we measured the miR‐125b levels in 20 ovarian cancer tissues and their corresponding normal tissues by qRT‐PCR assay. In ovarian cancer tissues, the miR‐125b level frequently declined and was negatively correlated with the LINC00152 expression (Figure 3B,C, *P* < .001). Second, we confirmed that silencing of miR‐125b significantly increased the LINC00152 level, whereas upregulation of miR‐125b greatly decreased the LINC00152 level (Figure 3D,E). Third, we performed a luciferase reporter assay and RNA‐binding protein immunoprecipitation (RIP) for direct evidence of the presence of miR‐125b bound to LINC00152. As shown in Figure 3F,G, co‐transfection with pre‐miR‐125b and LINC00152‐wt dramatically decreased the luciferase reporter activity compared to SKOV3 and A2780 cells that were cotransfected with WT vector and negative control or cotransfected with LINC00152‐mut vector and pre‐miR‐125b. MicroRNAs bind their targets and cause translational repression and/or RNA degradation in an AGO2‐dependent manner.^23^ The RIP experimental results verified that both miR‐125b and LINC00152 were found in the Ago2 pellet, indicating that they both existed in the RISC complex (Figure 3H).

**Figure 4:**
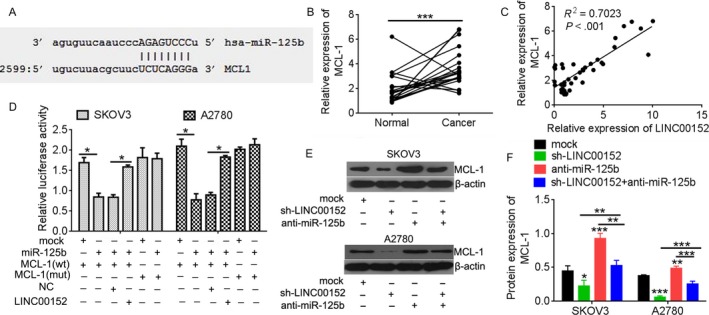
MCL‐1 was regulated by LINC00152 through competitive binding miR‐125b. A, Alignment of MCL‐1 sequences with miR‐125b as identified by Microrna (http://www.microrna.org/microrna). B, qRT‐PCR was performed to detect the expression of MCL‐1 in paired normal and ovarian cancer tissues (n = 20). C, The correlation between LINC00152 expression level and MCL‐1 level was measured in 20 ovarian cancer tissues (*P* < .001). D, The 3′‐UTR of MCL‐1 was fused to the luciferase coding region and cotransfected into SKOV3 and A2780 cells withmiR‐125b mimics to confirm that MCL‐1 is the target of miR‐125b. MCL‐1 3′‐UTR and miR‐125b mimics constructs were cotransfected into SKOV3 and A2780 cells with plasmids expressing LINC00152 or with a control vector, and the relative luciferase activity was determined at 48 h after transfection. E, Western blot analysis validated miR‐125b targets of Mcl‐1 after in SKOV3 and A2780 cells after transfected with sh‐LINC00152, or together with anti‐miR‐125b. β‐actin was used a loading control. F, Quantification analysis of MCL‐1 expression by IPP6.0. Error bars represent the mean + SD of triplicate experiments, *P <* *.05, **P <* *.01, ***P <* *.001

Because MCL‐1 is a direct target of miR‐125b,^14^ we further investigated the potential role of miR‐125b‐mediated regulation of MCL‐1 in ovarian cancer and its association with LINC00152. The binding site between miR‐125b and MCL‐1 was the same as for LINC00152 (Figure 4A). Moreover, MCL‐1 was overexpressed in ovarian cancer and positively correlated with LINC00152 levels (Figure 4B,C).

The results of luciferase activity assays showed that miR‐125b increased the activity of the wild‐type 3′‐UTR of MCL‐1, but not that of the mutant, confirming that MCL‐1 is a direct target and is upregulated by miR‐125b (Figure 4D). However, upregulation of LINC00152 suppressed the effect of miR‐125b for activation of the MCL‐1. To better understand the relation between miR‐125b and its target MCL‐1, the MCL‐1 level was assessed by Western blotting in SKOV3 and A2780 cells that were treated with miR‐125b inhibitor or sh‐LINC00152. The results showed that knockdown of LINC00152 inhibited the MCL‐1 level compared with the control group and was reversed by miR‐125b inhibitor (Figure 4E,F). Taken together, our results indicated that LINC00152 is a target of miR‐125b and modulates the MCL‐1 level.

### 3.4 LINC00152 modulates the effect of miR‐125b on cell growth

**Figure 5:**
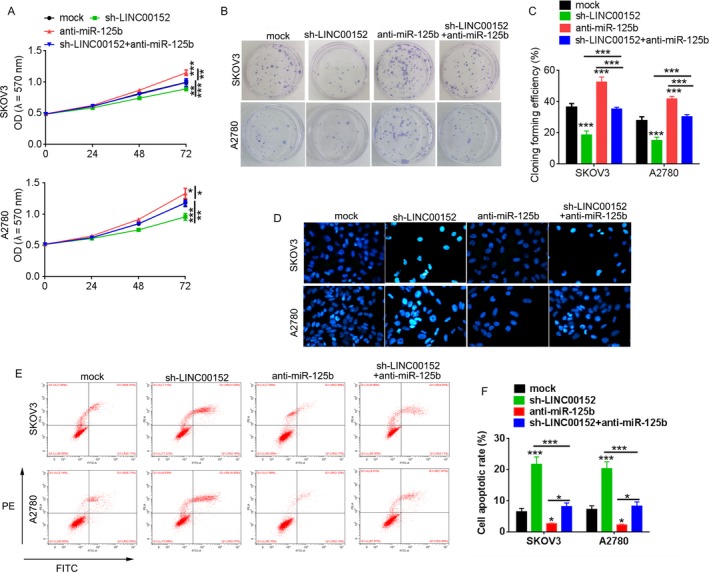
Knockdown of LINC00152 inhibits ovarian tumor growth in vitro. A, CCK8 assay was performed to detect cell proliferation in SKOV3 and A2780 cells after transfection with sh‐LINC00152 under the presence or absence of miR‐125b inhibitor. B, Colony‐forming unit assays. C, Quantification analysis of clone formation efficiency of (B). D, Hoechst 33258 stain assay detected the apoptosis cells. E, Flow apoptosis assay. F, Quantification of the apoptosis rate in (E). Error bars represent the mean ± SD of triplicate experiments, *P <* *.05, **P <* *.01, ***P <* *.001

miR‐125b was previously shown to promote cell growth progression.^18^ We therefore examined the effects of LINC00152 and miR‐125b on ovarian cancer by analyzing the effects of these molecules on cell growth progression. The results of MTT and colony‐forming assays showed that knockdown of LINC00152 inhibited miR‐125b‐induced cell growth in SKOV3 and A2780 cells (Figure 5A‐C). The results of flow cytometric analysis and Hoechst 33258 stain assay proved that downregulation of LINC00152 inhibited decreases in miR‐125b‐induced cell apoptosis (Figure 5D‐F).

**Figure 6:**
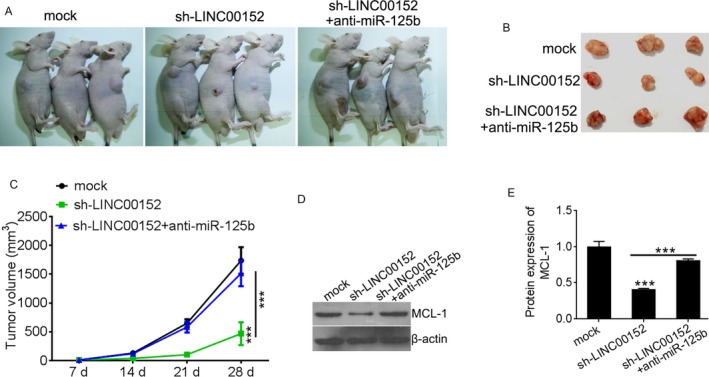
Knockdown of LINC00152 inhibits ovarian tumor growth in vivo. A, The SKOV3 cells transfected with sh‐LINC00152 alone or together with anti‐miR‐125b were injected into nude mice. The cells transfected with empty plasmid were used as negative control. B, The tumors obtained from mice on day 28 after injection. C, Tumor volumes were calculated every week. D, Representation of MCL‐1 expression in xenografts tumor using the Western blot method in sh‐NC or sh‐LINC00152 with or without anti‐miR‐125b group, respectively. E, Quantification analysis of MCL‐1 expression by IPP6.0. Error bars represent the mean ± SD of triplicate experiments, ***P <* *.001

To verify our in vitro findings, we established an in vivo xenograft model in nude mice. Compared with the NC control group, knockdown of LINC00152 significantly inhibited tumor growth. However, these inhibitory effects were reversed by miR‐125b downregulation (Figure 6A‐C). A Western blot assay was used to detect MCL‐1 levels in xenograft tumor tissues. Consistent with in vitro results, the MCL‐1 protein was inhibited by LINC00152 silencing, which was reversed by miR‐125b downregulation (Figure 6D,E). The results indicated that LINC00152 regulates the ovarian cancer growth via competitive binding of miR‐125b to MCL‐1.

### 3.5 LINC00152/miR‐125b/MCL‐1 axis modulated the mitochondrial apoptosis pathway on cell apoptosis in ovarian cancer cells

**Figure 7:**
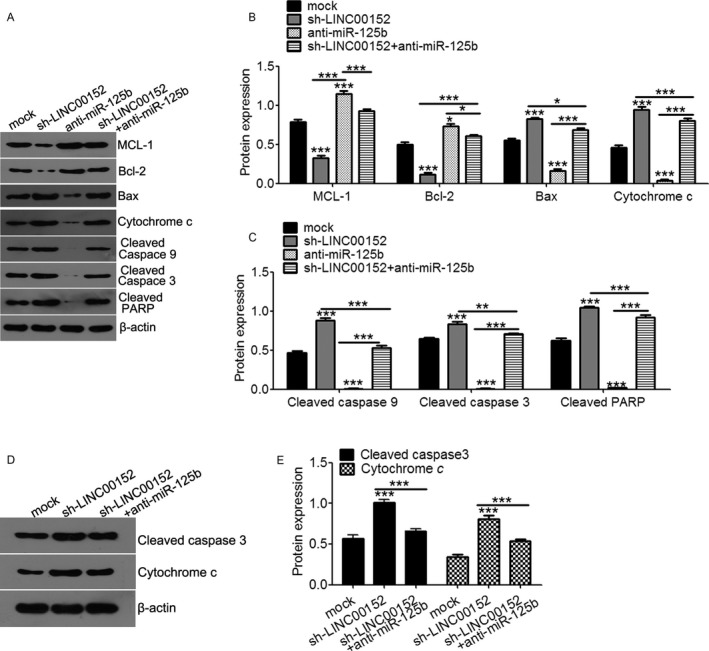
LINC00152 modulates the effect of miR‐125b on cell apoptosis protein expression. A, Western blot analysis of apoptosis protein expression in SKOV3 cells transfected with anti‐miR‐125b,sh‐LINC00152or both. B, Quantification analysis of MCL‐1, Bcl‐2, Bax, and cytochrome *c* expression in (A). C, Quantification analysis of cleaved caspase9, cleaved caspase3, and cleaved PARP level in (A). D, Representation of cleaved caspase3 and cytochrome *c* expression in xenografts tumor using Western blot method in sh‐NC, sh‐LINC00152 with or without anti‐miR‐125b group, respectively. E, Quantification analysis of cleaved caspase3 and cytochrome *c* expression. Error bars represent the mean ± SD of triplicate experiments, *P <* *.05, **P <* *.01, ***P <* *.001

Due to the importance of miR‐125b‐mediated mitochondrial pathways in cancer cell apoptosis, we further tested whether LINC00152 could regulate mitochondrial pathway‐related gene expressions. As shown in Figure 6A‐C, downregulation of LINC00152 decreased the expression of MCL‐1 and Bcl2, while increasing the expression of Bax, cytochrome *c*, cleaved caspase9, cleaved caspase 3, and cleaved PARP compared with the mock control. However, downregulation of miR‐125b reversed the regulation of LINC00152 to these target genes (Figure 7A‐C). In addition, concurrent silencing with LINC00152 and miR‐125b in vivo reduced the extent of cytochrome *c* and cleaved caspase 3 mediated by LINC00152 silencing alone (Figure 7D,E). These results revealed that the LINC00152/miR‐125b/MCL‐1 axis may modulate the mitochondrial apoptosis pathway during cell apoptosis.

**Figure 8:**
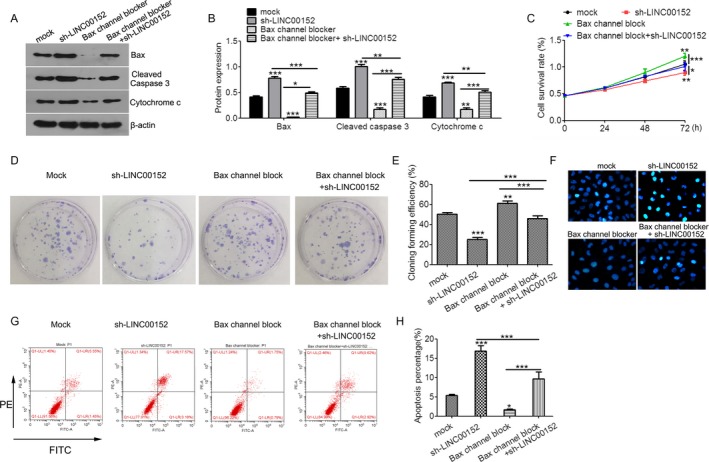
LINC00152 oncogenic activity is in part through regulation of mitochondrial apoptosis pathway. A, Western blot analysis of Bax, cleaved caspase3, and cytochrome *c* expression in SKOV3 cells treated with Bax channel block, sh‐LINC00152, or both. B, Quantification of Bax, cleaved caspase3, and cytochrome *c* expression in (A). C, CCK8 assay. D, Clone formation assay. E, Quantification analysis of clone formation efficiency of (D). F, Hoechst 33258 stain assay detected apoptotic cells. G, Flow apoptosis assay. H, Quantification of the apoptosis rate in (G). Error bars represent the mean ± SD of triplicate experiments, *P <* *.05, **P <* *.01, ***P <* *.001

Therefore, we used Bax channel blocker to block the mitochondrial apoptosis pathway and found that blockade of the mitochondrial apoptosis pathway reversed the extent of cytochrome *c* and cleaved caspase 3 induction by LINC00152 silencing in SKOV3 cells (Figure 8A,B). Furthermore, inhibition of the mitochondrial apoptosis pathway also restored LINC00152 silence‐mediated proliferative inhibition (Figure 8C), colony formation (Figure 8D,E), and cell apoptosis in SKOV3 cells (Figure 8F‐H). Taken together, these results indicated that LINC00152 antagonizes miR‐125b upregulation of MCL‐1expression to modulate the mitochondrial apoptosis pathway during ovarian cancer growth.

## 4 DISCUSSION

Increasing evidence supports that noncoding RNA plays an important regulatory role in the pathogenesis and progression of tumors.^24^ LINC00152 was overexpressed in several cancers, including esophageal squamous cell carcinoma, colorectal cancer, gastric cancer, and hepatocellular carcinoma.^25^ Increased LINC00152 levels might be potential biomarkers for predicting the poor progress of these cancers.^26^, ^27^, ^28^, ^29^, ^30^, ^31^ Mechanistically, LINC00152 overexpression dramatically promoted gallbladder cancer growth by regulating the phosphatidylinositol 3‐kinase (PI3K)/AKT signaling pathway.^9^ Chen et al revealed that LINC00152 was dramatically upregulated in gastric cancer and was positively correlated with tumor invasion, TNM stage, and poor survival. LINC00152 overexpression facilitated gastric cancer cell growth by accelerating the cell cycle through binding to the enhancer of zeste homolog 2 (EZH2) and decreasing p15 and p21 levels.^8^ LINC00152 could also accelerate gastric cancer cell proliferation through the EGFR‐mediated PI3K/AKT pathway.^32^, ^33^ In hepatocellular carcinoma, LINC00152 promoted cancer growth by activating the mechanistic target of the rapamycin (mTOR) pathway via binding to the promoter of EpCAM in a cis‐regulated manner.^10^ In this study, we first demonstrated that LINC00152 is markedly upregulated in ovarian cancer tissues, and the high level of LINC00152 is associated with a higher tumor histological grade, clinical stage, and low survival rate. Through knockdown of LINC00152 expression by shRNA, we observed its inhibitory effects on cell growth both in ovarian cancer cells and in a xenograft model. These results illustrate that LINC00152 functions as a tumor‐inducing lncRNA in ovarian cancer development.

Among the functional mechanisms of lncRNA, competing endogenous RNA theory has been verified by increasing evidence, which suggests that lncRNAs‐miRNA‐mRNA may participate in this regulatory circuitry.^34^ LINC00152 has been reported to act as an endogenous sponge of miR‐193a‐3a to confer oxaliplatin resistance in colon cancer.^26^ Therefore, we hypothesized that LINC00152 may also work as a ceRNA in ovarian cancer. We first predicted that LINC00152 was a potential target of miR‐125b, as the same binding site was also found in MCL‐1 and miR‐125b, which had a verified targeting relationship.^14^ LINC00152 was located in the cytoplasm, just as observed with miR‐125b and MCL‐1, which provides the necessary conditions for endogenous competition.^14^, ^35^ Second, we proved that miR‐125b was negatively correlated with LINC00152 in ovarian cancer tissues, while MCL‐1 was positively correlated. Either upregulation or silencing of the miR‐125b level could negatively affect LINC00152 levels in ovarian cancer cells. Furthermore, the results of the dual‐luciferase reporter assay and RNA‐binding protein immunoprecipitation assay indicated that LINC00152 was directly binding with miR‐125b and that overexpression of LINC00152 inhibited the direct binding between miR‐125b and MCL‐1. Above all, functional recovery experiments proved that silencing of LINC00152 resulted in a significant decrease in growth in ovarian cancer, and this regulation occurred via MCL‐1 and is abolished by miR‐125b inhibitors. Those studies supported the assumption that LINC00152 could work as a “molecular sponge” to compete for miR‐125b and to modulate their target MCL‐1.

MCL‐1, a pro‐apoptotic member of the Bcl‐2 family, is highly expressed in cancer and protects cells from apoptosis by binding to Bax and Bak, there by blocking mitochondrial outer membrane permeabilization, cytochrome *c* release, and the activation of the caspase cascade.^36^ In ovarian cancer, the MCL‐1 level upregulated expression and was positively associated with poor prognosis in ovarian cancer carcinomas.^37^ Silencing of MCL‐1 was key for promoting cell apoptosis in ovarian cells,^38^ which provided evidence to support the hypothesis that LINC00152 regulates MCL‐1 expression and inhibited mitochondrial‐dependent apoptosis to confer ovarian cancer growth. To investigate the miRNA‐related functions of LINC00152 that regulate mitochondrial‐dependent apoptosis in ovarian cancer pathogenesis, a functional recovery experiment was performed. The results of qRT‐PCR, Western blot, flow cytometric analysis, and Hoechst 33258 stain assay confirmed that LINC00152 knockdown could inhibit MCL‐1 expression and abolish the blockade of Bax channel that is induced by mitochondrial‐dependent cell death inhibition.

In conclusion, we demonstrated for the first time the oncogenic role of LINC00152 in ovarian cancer. An increased LINC00152 level is associated with poor prognosis for ovarian cancer patients. Silencing of LINC00152 dramatically declined ovarian cancer cell growth and caused apoptosis in vitro and in vivo by acting as a ceRNA target of miR‐125b to downregulate MCL‐1 expression and induce mitochondrial‐dependent cell death inhibition. Our study facilitates the understanding of LINC00152 function in the tumorigenesis of ovarian cancer and provides a novel diagnostic marker and therapeutic target for ovarian cancer treatment.

## CONFLICT OF INTEREST

The authors declare no conflict interest.
